# Data-driven normalization strategies for high-throughput quantitative RT-PCR

**DOI:** 10.1186/1471-2105-10-110

**Published:** 2009-04-19

**Authors:** Jessica C Mar, Yasumasa Kimura, Kate Schroder, Katharine M Irvine, Yoshihide Hayashizaki, Harukazu Suzuki, David Hume, John Quackenbush

**Affiliations:** 1 Department of Biostatistics, Harvard School of Public Health, 677 Huntington Avenue, Boston, Massachusetts 02115, USA; 2 RIKEN, Omics Science Center, Yokohama Institute, 1-7-22 Suehiro-cho, Tsurumi-ku, Yokohama, 230-0045, Japan; 3 Institute for Molecular Biosciences, University of Queensland, St Lucia, Brisbane QLD 4072, Australia; 4 Roslin Institute, University of Edinburgh, Roslin Midlothian EH25 9PS, Scotland, UK; 5 Department of Biostatistics and Computational Biology, Dana-Farber Cancer Institute, 44 Binney Street, Boston, Massachusetts 02115, USA; 6 Department of Cancer Biology, Dana-Farber Cancer Institute, 44 Binney Street, Boston, Massachusetts 02115, USA

## Abstract

**Background:**

High-throughput real-time quantitative reverse transcriptase polymerase chain reaction (qPCR) is a widely used technique in experiments where expression patterns of genes are to be profiled. Current stage technology allows the acquisition of profiles for a moderate number of genes (50 to a few thousand), and this number continues to grow. The use of appropriate normalization algorithms for qPCR-based data is therefore a highly important aspect of the data preprocessing pipeline.

**Results:**

We present and evaluate two data-driven normalization methods that directly correct for technical variation and represent robust alternatives to standard housekeeping gene-based approaches. We evaluated the performance of these methods against a single gene housekeeping gene method and our results suggest that quantile normalization performs best. These methods are implemented in freely-available software as an R package qpcrNorm distributed through the Bioconductor project.

**Conclusion:**

The utility of the approaches that we describe can be demonstrated most clearly in situations where standard housekeeping genes are regulated by some experimental condition. For large qPCR-based data sets, our approaches represent robust, data-driven strategies for normalization.

## Background

qPCR is widely accepted as the "gold standard" for analysis of gene expression. Recent technological advances have greatly expanded the total number of genes that can be analyzed in a single assay; qPCR experiments now regularly analyze "moderate" numbers of genes, in the range of fifty to a few thousand [1-3]. However, as the size of qPCR experiments has expanded, the need for effective data normalization techniques has become increasingly apparent. Normalization is the process of adjusting the relative expression measures between samples to compensate for various sources of variability in the assay and so to allow accurate comparisons of the results between different samples and conditions.

Nearly all normalization methods are based on the assumption that one or more genes are constitutively expressed at near-constant levels under all experimental conditions and the expression levels of all genes in a sample are adjusted to satisfy that assumption. The most widely used control genes are those selected from among an assumed set of "housekeeping" genes and typical selections include highly expressed transcripts such as glyceraldehyde-3-phosphate dehydrogenase (GAPDH), *β*-actin (ACTB), and 18S ribosomal RNA [4-8]. In most qPCR experiments, a single housekeeping gene is chosen and added to the collection of experimental target genes to be assayed for each sample. These control genes are then compared between samples, a sample-specific scaling factor is calculated to equalize their expression and applied to all genes in that sample. This approach has numerous limitations, not least of which is that many of the experimental conditions may alter the expression of the control genes and evidence shows that housekeeping genes may not always perform optimally [9,10].

More sophisticated normalization methods use multiple housekeeping genes where their respective measures are combined to represent a "virtual" housekeeping gene.[11]. While this approach is more robust than single-gene methods, it too is based on potentially unfounded assumptions about which genes are stably expressed. These genes still need to be pre-selected and incorporated into the experimental design without any apriori evidence supporting their use. Further, when working with limited quantities of RNA, such as from patient samples, this reduces the number of interesting genes whose expression can be assayed.

Improvements in qPCR technology have allowed significantly larger numbers of genes to be profiled simultaneously for each sample. This allows not only more experimental genes to be tested, but also provides an opportunity for a larger number of control genes, spanning a wide range of expression levels, to be used. However, these broad surveys also offer the possibility of introducing potential biases in normalization by using the data themselves to identify a set of appropriate controls. In many ways, this parallels widely used normalization methods developed for DNA microarray expression analysis, where data-driven methods have become the standard for most experimental designs. Here we present two normalization methods for high-throughput qPCR-based data adapted from those commonly applied in DNA microarray analysis: rank-invariant set normalization and quantile normalization [12,13]. As an example, we apply these methods to a high-throughput qPCR dataset from a time series experiment performed by the RIKEN Genome Exploration Research Group to study the temporal transcriptional response of macrophage-like human cells to phorbol myristate acetate (PMA) exposure for a set of 2,396 genes.

## Results

### Quantile Normalization Algorithm

Quantile normalization is one of the most widely used methods in the analysis of microarray experiments [14]. Quantile normalization assumes that the on average, the distribution of gene transcript levels within the cell remains nearly constant across samples, so that if the expression of one gene increases, that of another decreases. A quantile is a measure that lets us assess the degree of spread in a data set. Examples include percentiles, where the data are divided into 100 regular intervals, and quartiles which split data into quarters. For these, the lower quartile represents the 25th percentile which means that 25% of the data is lower than that particular value. Quantile normalization generalizes that approach to an *n*-fold partition of the data, where *n *is the number of data points, and assumes that the data for individual samples have the same overall rank-order quantile distribution. Quantile normalization then adjusts the overall expression levels so that the distribution for all samples is equal.

In high-throughput qPCR experiments, the number of genes assayed in each sample can exceed the capacity of a single microtiter plate so assays for one sample are often distributed across multiple PCR plates. Consequently, in normalizing data, one must also consider plate-specific effects that may introduce a bias. To correct for plate effects in raw qPCR data, one natural solution is to apply a quantile normalization approach where we make the assumption that the distribution of gene expression measures is the same across all plates for the same experimental condition. This assumption will be reasonable when the gene allocation was based on factors that are unrelated to their expected expression levels; for example, genes are randomly assigned or the assignment is based on an alphabetical ordering by gene name. By forcing the distribution from each plate to be equal we remove the variability associated with plate-specific effects in the data. Note that alternative solutions to this problem exist, namely one could take a linear model-based approach which incorporates a covariate term to explicitly account for plate-specific effects.

Quantile normalization proceeds in two stages. First, if samples are distributed across multiple plates, normalization is applied to all of the genes assayed for each sample to remove plate-to-plate effects by enforcing the same quantile distribution on each plate. Then, an overall quantile normalization is applied between samples, assuring that each sample has the same distribution of expression values as all of the other samples to be compared. A similar approach using quantile normalization has been previously described in the context of microarray normalization [12]. Briefly, our method entails the following steps:

1. qPCR data from a single RNA sample are stored in a matrix *M *of dimension *k *(maximum number of genes or primer pairs on a plate) rows by *p *(number of plates) columns. Plates with differing numbers of genes are made equivalent by padded plates with missing values to constrain *M *to a rectangular structure.

2. Each column is sorted into ascending order and stored in matrix *M'*. The sorted columns correspond to the quantile distribution of each plate. The missing values are placed at the end of each ordered column. All calculations in quantile normalization are performed on non-missing values.

3. The average quantile distribution is calculated by taking the average of each row in *M'*. Each column in *M' *is replaced by this average quantile distribution and rearranged to have the same ordering as the original row order in *M*. This gives the within-sample normalized data from one RNA sample.

4. Steps analogous to 1 – 3 are repeated for each sample. Between-sample normalization is performed by storing the within-normalized data as a new matrix *N *of dimension *k *(total number of genes, in our example *k *= 2,396) rows by *n *(number of samples) columns. Steps 2 and 3 are then applied to this matrix.

### Rank-Invariant Set Normalization Algorithm

The use of "housekeeping genes" in normalization is based on the assumption that one or more genes are expressed at near constant levels across all conditions assayed in a particular experiment. However, DNA microarray and high-throughput qPCR analyses have shown that many genes assumed to be constant in their expression can vary between conditions [10,15,16]. Rather than making a priori assumptions about which genes are expressed in such a manner, if one has a large enough dataset it is possible to identify invariant genes using the data itself. This is the premise behind rank-invariant set normalization that was first described by Tseng *et al.*[13] for DNA microarray data. In its original implementation the algorithm selected genes that remained rank-invariant across two conditions, i.e. when genes are ordered according to their expression levels, rank-invariant genes have the same rank order in both conditions. Here we describe an extension of this method for use on qPCR data with any number of experimental conditions or samples in which we identify a set of stably-expressed genes from within the measured expression data and then use these to adjust expression between samples. Briefly,

1. qPCR data from all samples are stored in matrix *R *of dimension *g *(total number of genes or primer pairs used for all plates) rows by *s *(total number of samples).

2. We first select gene sets that are rank-invariant across a single sample compared to a common reference. The reference may be chosen in a variety of ways, depending on the experimental design and aims of the experiment. As described in Tseng *et al.*[13], the reference may be designated as a particular sample from the experiment (e.g. time zero in a time course experiment), the average or median of all samples, or selecting the sample which is closest to the average or median of all samples. Genes are considered to be rank-invariant if they retain their ordering or rank with respect to expression across the experimental sample versus the common reference sample. We collect sets of rank-invariant genes for all of the *s *pairwise comparisons, relative to a common reference. We take the intersection of all *s *sets to obtain the final set of rank-invariant genes that is used for normalization.

3. Let *α*_*j *_represent the average expression value of the rank-invariant genes in sample *j*. (*α*_1_, ..., *α*_*s*_) then represents the vector of rank-invariant average expression values for all conditions 1 to *s*.

4. We calculate the scale factor *β*_*j *_for sample *j *where *β*_*j *_represents the ratio of the rank-invariant average expression value in the first sample versus sample *j*, i.e.  for *j *= 1 to *s*.

5. Finally, we normalize the raw data by multiplying each column *j *of R by the scale factor *β*_*j *_for *j *= 1 to *s*.

For our qPCR PMA time series data, we identified five rank-invariant genes to be used for normalization (Table 1). glyceraldehyde-3-phosphate dehydrogenase (GAPDH); enolase 1, (alpha) (ENO1); heat shock protein 90 kDa alpha (cytosolic), class B member 1 (HSP90AB1), ACTB, eukaryotic translation elongation factor 1 alpha 1 (EEF1A1). Of these, GAPDH and ACTB are oft-used control genes, it is not surprising to find they did not have highly variable expression profiles in this experiment. The identification of some of the other genes was more unexpected. HSPCB encodes a heat-shock protein HSP 90-beta, whilst EEF1A1 is involved in translation. The current version of the Gene Ontology used was released on 2008-01-15.

**Table 1:** GO Categories for the Rank-Invariant Genes

**Gene**	**Cellular Component**	**Molecular Function**	**Biological Processes**
GAPDH	Cytoplasm	Glyceraldehyde-3-phosphate dehydrogenase (phosphorylating) activity; protein binding	Glycolysis

ENO1	--	Phosphopyruvate hydratase activity	--

HSPCB	Cytoplasm	Nitric-oxide synthase regulator activity; nitric-oxide synthase regulator activity	Response to unfolded protein; positive regulation of nitric oxide biosynthetic process

ACTB	Cytoplasm; cytoskeleton	Protein binding; structural constituent of cytoskeleton	Cell motility

EEF1A1	Cytoplasm; eukaryotic elongation factor 1 complex	GTP binding; protein binding	Translational elongation

The GO categories are listed for the five genes in the rank-invariant set that were identified as reasonable controls to be used for normalization of the PMA dataset. The presence of GAPDH and ACTB as controls were not surprising for this dataset. The inclusion of some of the other categories heat-shock protein activity and translation, were more surprising.

The advantage of the rank-invariant approach becomes very clear when you consider that with a standard normalization method, we would only normalize the raw data based on GAPDH or ACTB expression. However, not only does our approach validate the assumption of near constant expression of GAPDH and ACTB for this experiment, it also provides other stable genes that can be used for a more robust normalization without the need for any a priori selection.

### An Example of Single Housekeeping Gene Normalization Using GAPDH

In order to compare our approaches with existing methods, we also performed normalization based on a single housekeeping gene, which for the PMA data set, was GAPDH. GAPDH was the only gene assigned to every plate and to take advantage of this design, we performed normalization in the following way:

1. Multiple GAPDH expression measures are averaged within each sample. Let *δ*_*j *_represent the average expression value of GAPDH in sample *j*. (*δ*_1_, ..., *δ*_*s*_) then represents the vector of average GAPDH expression values for all conditions 1 to *s*.

2. We calculate the scale factor *λ*_*j *_for sample *j *where *λ*_*j *_represents the ratio of the GAPDH expression in the first sample versus sample *j*, i.e.  for *j *= 1 to *s*.

3. Finally, we normalize the raw data by multiplying the vector of data from sample *j *with the scale factor *λ*_*j *_for *j *= 1 to *s*.

The approach adopted represents an alternative version from how housekeeping gene normalization is typically performed in qPCR experiments. The standard approach taken is the delta-delta Ct method which involves two subtractions, first between the housekeeping gene expression value (in this case GAPDH) from the gene of interest measured in the control sample and second, the gene of interest measured in the experimental sample and the housekeeping gene in the experimental sample. We took this approach to exploit the design which involved GAPDH measurements being available for all plates in this data set. In doing so, our implementation of the housekeeping gene more closely parallels the other two methods.

### Comparison of Different Normalization Approaches

**Figure 1:**
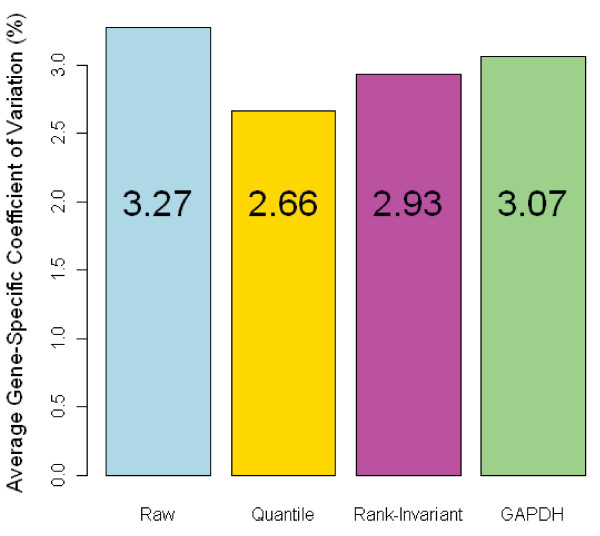
**Coefficient of Variation for Different Normalized Data Sets**. The CV values for the three different normalization methods on the PMA dataset are represented here in a barchart. The CV for the non-normalized (raw) dataset is included as a reference. The quantile method is associated with the lowest CV, implying the greatest reduction in technical variation in the data.

The PMA time series experiment described previously provided an opportunity to assess the performance of these methods relative to the use of the single housekeeping gene approach. The data was normalized using all three methods and the average gene-specific coefficient of variation (CV) was calculated to assess the overall reduction in technical noise associated with each approach. The CV measures the ratio of the standard deviation to the mean and captures the level of dispersion in the data. Therefore, a normalization method that better reduces technical noise will have a lower average CV. The results are shown in Figure 1. As can be seen, quantile normalization produces the lowest average CV (3.36%). Both the rank-invariant set method and the GAPDH have very similar average CV values (3.59, 3.60% respectively), while the CV for the non-normalized data was 4.77%. These results suggest that the quantile method has an advantage over the other normalization methods.

**Figure 2:**
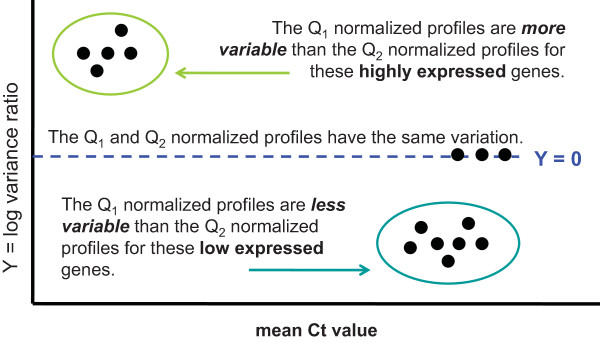
**Exemplar graph to clarify the interpretation of Figure 3**. The graph presents a visual pairwise comparison between two normalization algorithms Q_1 _and Q_2 _on the same data set. For each gene, we calculate the variance of its Q_1_-normalized expression profile and its Q_2_-normalized expression profile and plot the log_2_-ratio of this variance on the y-axis where Y = log_2 _[Q_1_-normalized: Q_2_-normalized]. A gene's log variance ratio is plotted against its expression (mean Ct value) on the x-axis. The regions where the data points fall in the graph give us an indication of which normalization algorithm produces noisier data and whether there is a differential bias in expression for genes most affected by this noise.

**Figure 3:**
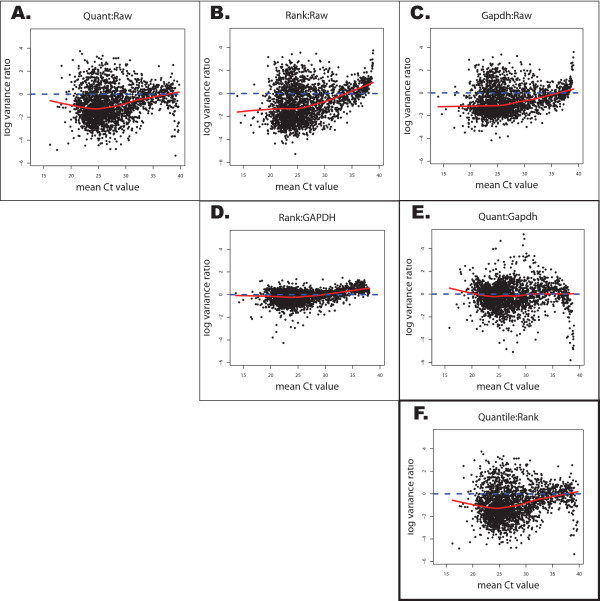
**Pairwise Comparisons of Different Normalized Data Sets**. Pairwise comparisons between the three different normalization methods and the non-normalized dataset. The graphs represent the log variance ratios for each gene versus its average Ct value. The red line is the smoothed lowess curve that captures the overall trend of the data in the plot. The dotted blue line represents horizontal axis. The direction of the ratio is reflected in each individual figure title, e.g. the ratios in Figure 3.3A are constructed by taking the log_2 _transformation of the GAPDH-normalized variance divided by the non-normalized variance for each gene. Points below the dotted blue line correspond to those genes where single gene GAPDH normalization has resulted in a greater reduction in variance relative to the variance of these genes in the non-normalized data.

We also examined the variance of the raw and normalized data as a function of expression level. Let *Q*_1 _and *Q*_2 _represent two normalization algorithms (e.g. *Q*_1 _= quantile). We calculate the variance of each gene expression profile normalized under *Q*_1 _and *Q*_2 _and plot the log_2_-transformed ratio of these variances as a function of the average expression of each gene for all genes. The red line represents a smoothed lowess curve that has been fitted to reflect the overall trend of the data [17]. When the curve drops below Y = 0 (the dotted blue line in Figures 2 and 3) we know that method *Q*_1 _effects a greater reduction in the variation of the data relative to method *Q*_2_. Similarly, when the red curve is above Y = 0, method *Q*_2 _is more effective in reducing the variation. If the data from both methods have similar variances then the red curve should remain at Y = 0. Bolstad *et al.*[12] originally used these plots for variance comparisons of different normalization methods for high density oligonucleotide array data.

Figure 3F shows a comparison of the data for the quantile and rank-invariant methods. The fact that the curve is below the horizontal axis for most of the expression spectrum (20 < Ct values < 37) indicates that quantile normalization generally produces a smaller variance than the rank-invariant method, independent of expression level. Comparing both data-driven approaches with the single housekeeping gene method (Figures 3D and 3E) indicates that both data-driven methods offer a slight advantage. In Figure 3E, we see that comparisons between quantile normalization and GAPDH normalization actually produce quite similar amounts of variability. It appears that for highly expressed genes, quantile normalization induces more noise than the GAPDH method however this is difficult to ascertain since there is sparse data at this end of the spectrum which reduces the accuracy of the lowess curve fitting. The rank-invariant method may be inferior for genes expressed at extremely low levels (large Ct values), although expression measures for such genes are generally thought to be unreliable.

Comparisons of normalized to non-normalized data (Figures 3A, 3B, 3C) also support considerable variance reductions associated with both data-driven approaches across the detectable range of qPCR. When compared to the non-normalized data, the quantile method has reduced variance across the entire expression spectrum. Both GAPDH and the rank-invariant set normalization had regions where these methods resulted in higher variation than the raw data. These regions however correspond to very low expressed genes (Ct > 35) and the data quality at this end of the spectrum is usually considered to be very poor. These plots demonstrate that the quantile method is more effective in reducing the variances of genes with expression levels that span the detectable range of qPCR compared to the other normalization methods.

As an example of how our normalization strategies perform in action, we highlighted the effects the different normalization methods had on four individual genes: E2F transcription factor 1 (E2F1), early growth response 1 (EGR1), v-myb myeloblastosis viral oncogene homolog (avian) (MYB), tumor necrosis factor, alpha-induced protein 3 (TNFAIP3). These genes were selected as examples because they were typical genes of interest for PMA-activated THP-1 cells. Comparing the variances of each profile (see Additional files 1 and 2) reveals that in all four cases, the lowest variances are associated with the data-driven approaches. It is also interesting to note that for the E2F1, EGR1 and TNFAIP3 expression profiles, the GAPDH normalization method results in profiles with slightly higher variance than the non-normalized profile.

### Implementation

Both quantile and rank-invariant set normalization algorithms for qPCR data are implemented as freely available, open source software using the statistical computing language R. We have adapted the quantile normalization algorithm that originally was included in the limma package [18]. Our software is distributed as an R package called qpcrNorm and a short tutorial outlining its use is available from [19] (see Additional file 3). The qpcrNorm package is also freely available from Bioconductor [20].

## Discussion

As high-throughput qPCR has become more widespread, it has become clear that we need more effective data methods to ensure the consistent acquisition of reliable, high quality results. Here, we present two data-driven normalization methods for qPCR that offer significant advantages. Rank-invariant normalization eliminates the need for possibly un-founded assumptions about which genes will not be differentially regulated in an experiment while relying on a generally accepted approach. Quantile normalization extends this by allowing for correction of plate effects.

Both the rank-invariant set and single housekeeping gene approaches are examples based on scaling. The raw expression values are transformed by an empirically-derived scale factor and consequently we see an overall reduction in the variability of the normalized data. On the other hand, the quantile approach replaces the raw data with representative values derived from the average quantile data distribution. As a result, we see the variability either preserved or increased in the normalized data. These effects are noticeably obvious by comparing the tightness of the graphs in Figure 3D versus 3E (also seen by comparing Figures 3A, B, D to Figures 3C, E, F).

Not only does quantile normalization tend to increase the variability in the distribution, we also notice an increase in the number of genes with more upper extreme Ct values. This is reflected by the tailing effect, as observed in Figure 3C, where more genes have values that all fall close to the maximum Ct value of 40 cycles. Looking at the empirical distributions (Figure 3), we see that the quantile normalized data has the heaviest right tail out of the three normalized data sets. Also of interest is the fact that the quantile normalization method appears to preserve the original distribution of the raw data.

Our methods are specifically designed for high-throughput data sets where the number of genes or primer pairs used in the qPCR experiment is moderate to large (greater than 50). We expect the robustness of both the rank-invariant gene method and the quantile method to break down when the number of genes or primer pairs decreases and drops below this threshold. We were unable to assess this quantitatively however due to the lack of available data.

The quantile method first focuses on the data within a sample, and applies a normalization correction to ensure the quantile distribution from different plates for the sample have the same quantile distribution. If there were a small number of genes or primer pairs assigned to each plate, then this normalization would be more susceptible to outliers. It is assumed that when there is a large number of genes or primer pairs, that the corresponding quantile distribution will be reasonably smooth and cover a realistic range of values expected for qPCR data. When the number of genes shrinks, the validity of this assumption may become questionable.

## Conclusion

The data-driven normalization alternatives that we have presented have clear advantages when widely-used housekeeping genes are regulated by some experimental factor or condition. Vandesompele *et al.*[11] use a panel of ten common housekeeping genes and advocate using at least three of these genes for normalization of a given experimental design. However the appropriateness of this approach still hinges heavily on the assumption that any of these genes is not regulated in the experiment and consequently is suitable as a control. In the presence of large amounts of data, our data-driven normalization methods represent a robust approach since no a priori assumptions are made regarding which genes might be used as controls and, in general, provides many more genes for normalization than Vandesompele *et al. *and colleagues suggest. In the case of rank-invariant set normalization, genes which do satisfy the properties of being a good control gene are easily identified. Quantile normalization corrects for plate-specific effects in the data by requiring samples to have similar distributions, although it does not have the aesthetic advantage of identifying control genes. Overall, our analyses indicate that both methods outperform approaches using a priori sets of housekeeping genes and that quantile normalization gives the best overall performance. Although we used a time course experiment to test these normalization approaches, these methods are applicable to any high-throughput qPCR setup.

## Methods

### qPCR Gene Expression Data

The THP-1 cell line was sub-cloned and one clone (#5) was selected for its ability to differentiate relatively homogeneously in response to phorbol 12-myristate-13-acetate (PMA) (Sigma). THP-1.5 was used for all subsequent experiments. THP-1.5 cells were cultured in RPMI, 10% FBS, Penicillin/Streptomycin, 10 mM HEPES, 1 mM Sodium Pyruvate, 50 uM 2-Mercaptoethanol. THP-1.5 were treated with 30 ng/ml PMA over a time-course of 96 h. Total cell lysates were harvested in TRIzol reagent at 1, 2, 4, 6, 12, 24, 48, 72, 96 hours, including an undifferentiated control. Undifferentiated cells were harvested in TRIzol reagent at the beginning of the LPS time-course. One biological replicate was prepared for each time point. Total RNA was purifed from TRIzol lysates according to manufacturer's instructions. Gene-specific primer pairs were designed using Primer3 software [21], with an optimal primer size of 20 bases, amplification size of 140 bp, and annealing temperature of 60°C. Primer sequences were designed for 2,396 candidate genes including four potential controls: GAPDH, beta actin (ACTB), beta-2-microglobulin (B2M), phosphoglycerate kinase 1 (PGK1). The RNA samples were reverse transcribed to produce cDNA and then subjected to quantitative PCR using SYBR Green (Molecular Probes) using the ABI Prism 7900 HT system (Applied Biosystems, Foster City, CA, USA) with a 384-well amplification plate; genes for each sample were assayed in triplicate. Reactions were carried out in 20 *μ*L volumes in 384-well plates; each reaction contained: 0.5 U of HotStar Taq DNA polymerase (Qiagen) and the manufacturer's 1× amplification buffer adjusted to a final concentration of 1 mM MgCl_2_, 160 *μ*M dNTPs, 1/38000 SYBR Green I (Molecular Probes), 7% DMSO, 0.4% ROX Reference Dye (Invitrogen), 300 nM of each primer (forward and reverse), and 2 *μ*L of 40-fold diluted first-strand cDNA synthesis reaction mixture (12.5 ng total RNA equivalent). Polymerase activation at 95°C for 15 min was followed by 40 cycles of 15 s at 94°C, 30 s at 60°C, and 30 s at 72°C. The dissociation curve analysis, which evaluates each PCR product to be amplified from single cDNA, was carried out in accordance with the manufacturer's protocol. Expression levels were reported as Ct values.

The large number of genes assayed and the replicates measures required that samples be distributed across multiple amplification plates, with an average of twelve plates per sample. Because it was envisioned that GAPDH would serve as a single-gene normalization control, this gene was included on each plate. All primer pairs were replicated in triplicates.

Raw qPCR expression measures were quantified using Applied Biosystems SDS software and reported as Ct values. The Ct value represents the number of cycles or rounds of amplification required for the fluorescence of a gene or primer pair to surpass an arbitrary threshold. The magnitude of the Ct value is inversely proportional to the expression level so that a gene expressed at a high level will have a low Ct value and vice versa.

Replicate Ct values were combined by averaging, with additional quality control constraints imposed by a standard filtering method developed by the RIKEN group for the preprocessing of their qPCR data. Briefly this method entails:

1. Sort the triplicate Ct values in ascending order: *Ct*_1_, *Ct*_2_, *Ct*_3_. Calculate differences between consecutive Ct values: *difference*_1 _= *Ct*_2 _- *Ct*_1 _and *difference*2 = *Ct*_3 _- *Ct*_2_.

2. Four regions are defined (where Region_4 _overrides the other regions):

Region_1_: difference ≤ 0.2

Region_2_: 0.2 < difference ≤ 1.0

Region_3_: 1.0 < difference

Region_4_: one of the Ct values in the difference calculation is 40

If *difference*_1 _and *difference*_2 _fall in the same region, then the three replicate Ct values are averaged to give a final representative measure. If *difference*_1 _and *difference*_2 _are in different regions, then the two replicate Ct values that are in the small number region are averaged instead.

This particular filtering method is specific to the data set we used here and does not represent a part of the normalization procedure itself; Alternate methods of filtering can be applied if appropriate prior to normalization. Moreover while the presentation in this manuscript has used Ct values as an example, any measure of transcript abundance, including those corrected for primer efficiency can be used as input to our data-driven methods.

### Data access and availability

All data analyzed in this manuscript have been made publicly available through GEO (accession number: GSE15528).

## Authors' contributions

JCM and JQ conceived of the study and the overall approach and were primary authors of the manuscript. JCM developed and tested the algorithms and evaluated the results and created the R package. KS and DH designed the laboratory experiments and KS generated the RNA samples. YK, KMI, HS, and YH and were responsible for designing and conducting the qPCR experiments; YK also contributed to testing and evaluating the normalization algorithms.

All authors read and approved the final manuscript.

## Supplementary Material

Additional file 1 **Figure S1**. Expression Profiles for Four Genes Normalized by Different approaches. Click here for file

Additional file 2 **Table S1**. Variances of 4 Gene Expression Profiles Normalized by Different Approaches. Click here for file

Additional file 3 **Tutorial and qpcrNorm Software**. The R package qpcrNorm and a tutorial outlining its use. Click here for file

## References

[B1] Arany ZP, Haines JL et al (2008). High-throughput quantitative real-time PCR. Current Protocols in Human Genetics.

[B2] VanGuilder HD, Vrana KE, Freeman WM (2008). Twenty-five years of quantitative PCR for gene expression analysis. Biotechniques.

[B3] Spurgeon SL, Jones RC, Ramakrishnan R (2008). High throughput gene expression measurement with real time PCR in a microfluidic dynamic array. PLoS ONE.

[B4] Bustin SA, Gyselman VG, Williams NS, Dorudi S (1999). Detection of cytokeratins 19/20 and guanylyl cyclase C in peripheral blood of colorectal cancer patients. British Journal of Cancer.

[B5] Hamalainen HK, Tubman JC, Vikman S, Kyrola T, Ylikoski E, Warrington JA, Lahesmaa R (2001). Identification and validation of endogenous reference genes for expression profiling of T helper cell differentiation by quantitative real-time RT-PCR. Anal Biochem.

[B6] Kreuzer KA, Lass U, Landt O, Nitsche A, Laser J, Ellerbrok H, Pauli G, Huhn D, Schmidt CA (1999). Highly sensitive and specific fluorescence reverse transcription-PCR assay for the pseudogene-free detection of beta-actin transcripts as quantitative reference. Clinical Chemistry.

[B7] Oliveira JG, Prados RZ, Guedes AC, Ferreira PC, Kroon EG (1999). The housekeeping gene glyceraldehyde-3-phosphate dehydrogenase is inappropriate as internal control in comparative studies between skin tissue and cultured skin fibroblasts using Northern blot analysis. Archives of Dermatological Research.

[B8] Tricarico C, Pinzani P, Bianchi S, Paglierani M, Distante V, Pazzagli M, Bustin SA (2002). Quantitative real-time reverse transcription polymerase chain reaction: normalization to rRNA or single housekeeping genes is inappropriate for human tissue biopsies. Anal Biochem.

[B9] Schmittgen TD, Zakrajsek BA (2000). Effect of experimental treatment on housekeeping gene expression: validation by real-time, quantitative RT-PCR. J Biochem Biophys Methods.

[B10] Thellin O, Zorzi W, Lakaye B, De Borman B, Coumans B, Hennen G, Grisar T, Igout A, Heinen E (1999). Housekeeping genes as internal standards: use and limits. J Biotechnol.

[B11] Vandesompele J, de Preter K, Pattyn F, Poppe B, van Roy N, de Paepe A, Speleman F (2002). Accurate normalization of real-time quantitative RT-PCR data by geometric averaging of multiple internal control genes. Genome Biology.

[B12] Bolstad BM, Irizarry RA, Astrand M, Speed TP (2003). A comparison of normalization methods for high density oligonucleotide array data based on bias and variance. Bioinformatics.

[B13] Tseng GC, Oh MK, Rohlin L, Liao JC, Wong WH (2001). Issues in cDNA microarray analysis: quality filtering, channel normalization, models of variations and assessment of gene effects. Nucleic Acids Research.

[B14] Grewal A, Lambert P, Stockton J, Baxevanis AD et al (2007). Analysis of Expression Data: An Overview. Current Protocols in Bioinformatics.

[B15] Suzuki T, Higgins PJ, Crawford DR (2000). Control selection for RNA quantitation. Biotechniques.

[B16] Warrington JR, Nair A, Mahadevappa M, Tsyganskaya M (2000). Comparison of human adult and fetal expressin and identification of 535 housekeeping/maintenance genes. Physiol Genomics.

[B17] Cleveland W (1979). Robust locally weighted regression and smoothing scatterplots. J Am Stat Assoc.

[B18] Smyth GK, Gentleman R, Carey V, Dudoit S, Irizarry R, Huber W (2005). Limma: linear models for microarray data. Bioinformatics and Computational Biology Solutions using R and Bioconductor.

[B19] Supplemental tutorial to accompany the R package. http://compbio.dfci.harvard.edu/pubs/qpcrNorm_supplemental.zip.

[B20] Bioconductor. http://www.bioconductor.org.

[B21] Rozen S, Skaletsky H (2000). Primer3 on the WWW for general users and for biologist programmers. Methods Mol Biol.

