# Supplementary material for: Data-driven normalization strategies for high-throughput quantitative RT-PCR
Source: BMC Bioinformatics. 2009 Apr 19;10:110. doi: 10.1186/1471-2105-10-110 (PMC2680405; doi:10.1186/1471-2105-10-110)
Supplement: Additional file 2 — Table S1. Variances of 4 Gene Expression Profiles Normalized by Different Approaches. [file 1471-2105-10-110-S2.doc]

## Supplementary Table 1 – Variances of 4 Gene Expression Profiles Normalized by Different Approaches

The table reports the variances calculated for each profile of Ct values, as normalized by each different method for each of the four example genes.

| **Gene** | **Normalization Method** | | | |
| --- | --- | --- | --- | --- |
|  | *Raw* | *Gapdh* | *Rank Invariant* | *Quantile* |
| **E2F1** | 1.363 | 1.422 | 0.614 | 0.704 |
| **EGR1** | 6.435 | 6.498 | 4.125 | 2.660 |
| **MYB** | 4.358 | 2.829 | 1.478 | 0.696 |
| **TNFAIP3** | 1.941 | 2.026 | 1.130 | 1.540 |
