# Supplementary material for: Data-driven normalization strategies for high-throughput quantitative RT-PCR
Source: BMC Bioinformatics. 2009 Apr 19;10:110. doi: 10.1186/1471-2105-10-110 (PMC2680405; doi:10.1186/1471-2105-10-110)
Supplement: Additional file 3 — Tutorial and qpcrNorm Software. The R package qpcrNorm and a tutorial outlining its use. [file 1471-2105-10-110-S3.zip › qpcrNorm_supplemental/qpcrNorm.pdf]

Applying Data-Driven Normalization Strategies for qPCR Data Using Bioconductor

Jessica Mar

April 3, 2009

1 Introduction

High-throughput real-time quantitative reverse transcriptase polymerase chain reaction (qPCR) is a widely used technique in experiments where expression patterns of genes are to be profiled. qPCR is widely accepted as the "gold standard" for analysis of gene expression. Recent technological advances have greatly expanded the total number of genes that can be analyzed in a single assay; qPCR experiments now regularly analyze "moderate" numbers of genes, in the range of fifty to a few thousand [1-3]. However, as the size of qPCR experiments has expanded, the need for effective data normalization techniques has become increasingly apparent. Normalization is the process of adjusting the relative expression measures between samples to compensate for various sources of variability in the assay and so to allow accurate comparisons of the results between different samples and conditions.

This short vignette demonstrates how to use the functions available in the package `qpcrNorm`. As an example, we apply these functions to an artifically generated qPCR data set. This data has been closely simulated from original qPCR data, there are Ct measures for 2396 genes on samples taken at 13 times points. Each measurement was replicated three times and each sample was split over multiple 384-well plates. This data is stored as `qpcrBatch.object`.

```
> library(qpcrNorm)
> data(qpcrBatch.object)
```

Data objects in the `qpcrNorm` package make use of the S4 class `qpcrBatch`. The example data `qpcrBatch.object` is stored as a `qpcrBatch` object. This class has been designed to facilitate the storage and analysis of qPCR data in R, and in short, make life a bit easier for users of `qpcrNorm`. The `qpcrBatch` object is similar to other Bioconductor class representations `AffyBatch` and `ExpressionSet`.

```
> slotNames(qpcrBatch.object)
```

```
[1] "geneNames"  "plateIndex" "exprs"      "normalized" "normGenes"
```

In addition to gene names, the `qpcrBatch` class requires a set of indices to denote which genes or primer pairs belong to the same plate. This information is critical to the quantile normalization method. The class also contains a `normalized` logical flag so the user can keep track of which data object has been normalized already. A slot for the genes which were involved in the normalization (e.g. rank-invariant set normalization) lets the user refer back to these, either for reporting purposes or for further analysis.

2 Preprocessing Raw qPCR Data

Raw qPCR data is often report as Ct values. The Ct value represents the number of cycles or rounds of amplification required for the fluorescence of a gene or primer pair to surpass an arbitrary threshold. The magnitude of the Ct value is inversely proportional to the expression level so that a gene expressed at a high level will have a low Ct value and vice versa.

Replicate Ct values can be combined, either by straightforward averaging or are sometimes subjected to a quality control (QC) filter prior to averaging. Our collaborators apply a QC filter based on a requirement that all three requirements be ”similar” to each other, in order to be included in the final average. This filter is implemented in the function `ctQc`. Please note that this filter method was used by our collaborators and does not represent a part of our normalization procedure itself. Alternate methods of filtering can be applied if appropriate prior to normalization. Moreover while we have used Ct values as an example here, any measure of transcript abundance, including those corrected for primer efficiency can be used as input to our data-driven methods.

See `readQpcr` and `readQpcrBatch` for info on how to get raw data from a text file into R.

3 Normalizing qPCR Data

Three normalization algorithms are packaged in `qpcr`.

- rank-invariant set normalization
- quantile normalization
- normalization based on housekeeping genes.

The rank-invariant set normalization algorithm first identifies which set of genes remain rank-invariant (meaning their ordering based on gene expression doesn’t change) across multiple pairwise comparisons. Usually these comparisons involve comparing the experimental sample against a baseline, either the first sample (as in time zero, in a time course) or an average of all samples. This algorithm was first described by Tseng et al.

(2001) for the normalization of cDNA microarray data. We have adapted this algorithm to be used for high-throughput qpcr data.

The average expression of the genes in this rank-invariant set are combined to form a scale factor which is used to normalize the qPCR data set.

Applying rank-invariant set normalization, using the first sample as the baseline:

```
> mynormRI.data <- normQpcrRankInvariant(qpcrBatch.object, 1)
```

The genes that were in the rank-invariant set:

```
> mynormRI.data@normGenes
```

```
[1] "Syt3"   "Tysnd1"
```

The quantile normalization algorithm works on the premise that samples should have expression measures with approximately the same distribution. The algorithm also assumes that within each sample, the distributions should be the same across multiple plates. This algorithm was first described by Bolstad et al. (2003) in the context of normalizing microarray data. We've adapted the algorithm to be used for qpcr data.

```
> mynormQuant.data <- normQpcrQuantile(qpcrBatch.object)
```

Using either a single housekeeping gene or panel of controls is the most common normalization method for qPCR data. The `normQpcrHouseKeepingGenes` function allows normalization to be performed using either one or more houskeeping genes. (Since this is an artificial data set, the gene Gpx4 was chosen at random and is used purely as a means to demonstrate this function).

Please note that our implementation of housekeeping gene-based normalization deviates from the standard approach taken, which is usually the delta-delta Ct method. This method involves two subtractions, first between the housekeeping gene expression value (e.g. GAPDH) from the gene of interest measured in the control sample and second, the gene of interest measured in the experimental sample and the housekeeping gene in the experimental sample.

Our housekeeping-gene normalization approach is based on scaling all measurements to the housekeeping gene profile. Please see Mar et al. (2009) for more details on how this approach works. The advantage of this approach is that it parallels more closely the high-throughput nature of the other two algorithms we have implemented.

```
> mynormHK.data <- normQpcrHouseKeepingGenes(qpcrBatch.object,
+     c("Gpx4"))
```

We can also use the generic function `normalize` to normalize qpcr data stored in a `qpcrBatch` object.

```
> mynormQuant.data <- normalize(qpcrBatch.object, "quantile")
> mynormHK.data <- normalize(qpcrBatch.object, "housekeepinggenes",
+     c("Gpx4"))
```

4 Assessing Performance

We can see how sample to sample variability has been reduced (or in this case removed).

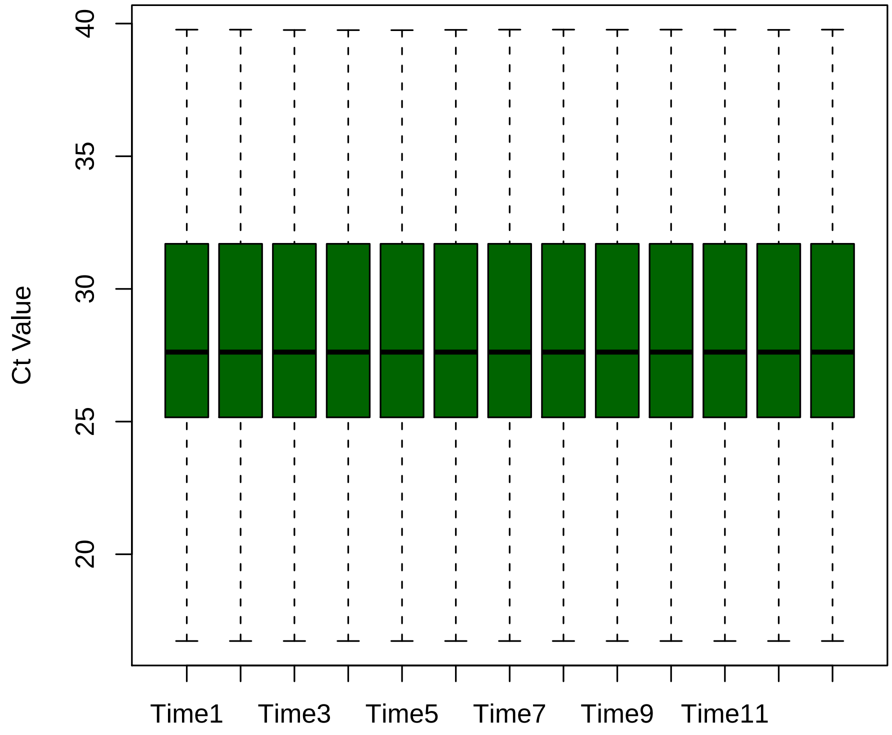

Figure 1: Sample Distributions of Quantile-Normalized Data

We can look at the histograms of the normalized data to get a sense of how the different methods have affected the original data. The data normalized by the data-driven methods retain most of the original distribution shape. The housekeeping-gene normalization has reduced the number of extreme Ct values (these correspond to genes with very low expression).

We can also calculate the coefficient of variation (CV) in the resulting normalized data. With this data set, we don't see a lot of difference among the CV values for the three normalization methods.

```
> cvVals <- c(calcCV(qpcrBatch.object), calcCV(mynormHK.data),
+     calcCV(mynormQuant.data), calcCV(mynormRI.data))
```

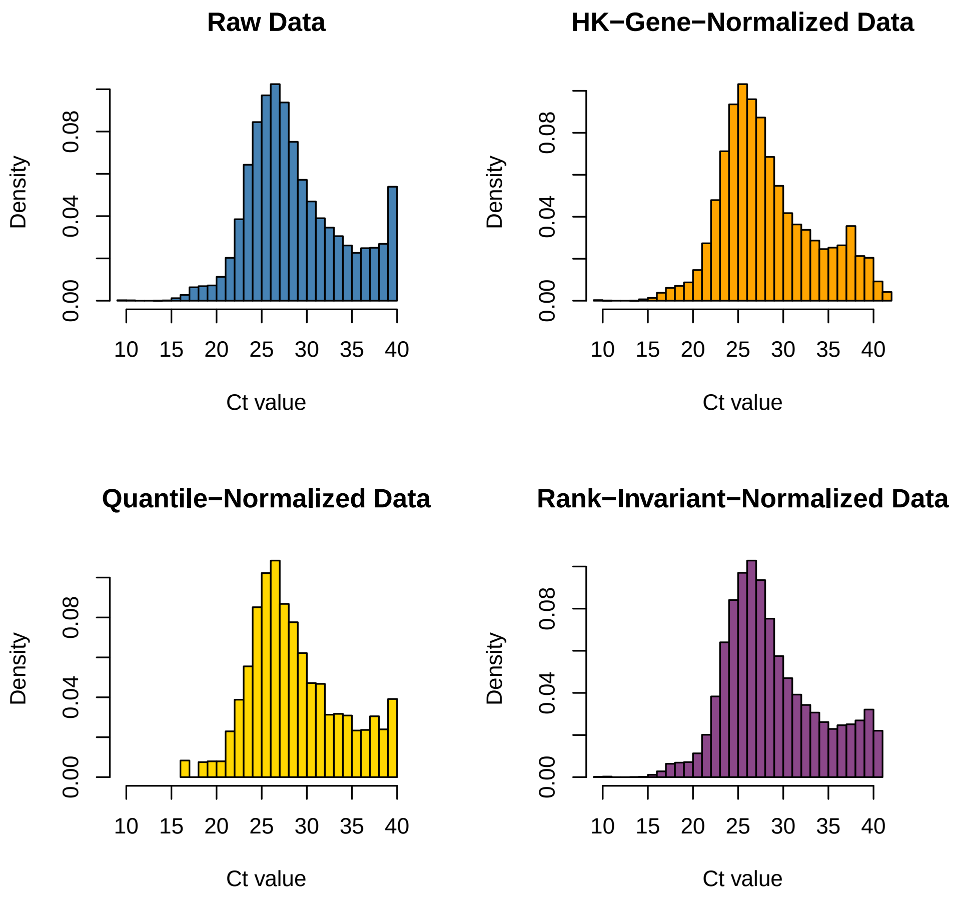

Figure 2: Histograms of Data Normalized by Different Methods

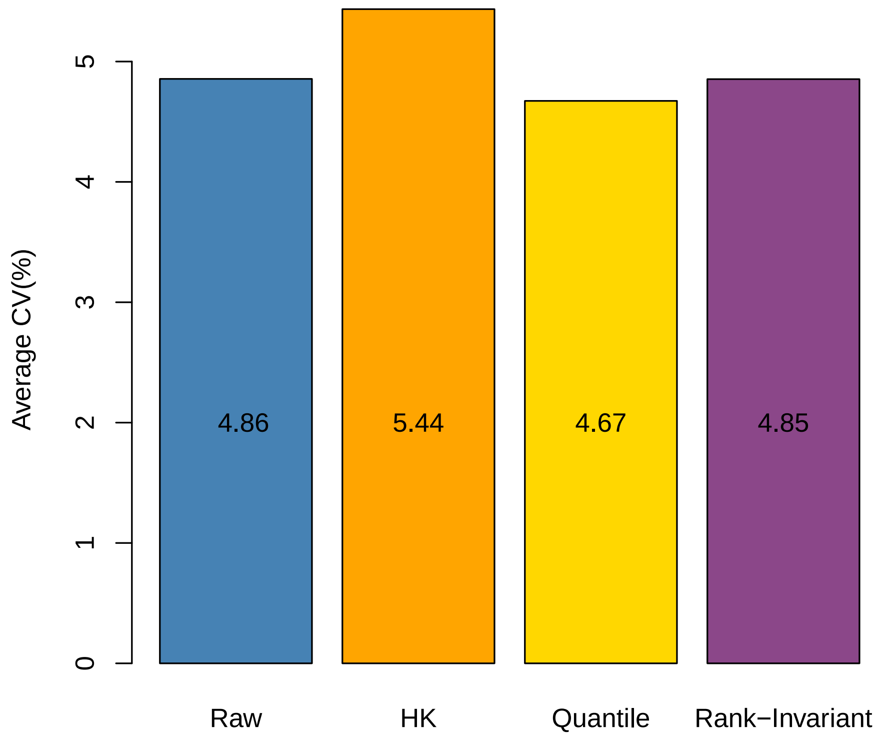

Figure 3: Barchart of Average CV Values

The `plotVarMean` function provides a means to directly compare the effects of two normalization methods on the same data set. This plot displays the `log`-base 2 ratio Let `Gij` be the variance of the expression values of gene `i` that have been normalized with method `j`. We plot the `log`2-transformed ratio of `Gij` to `Gik` as a function of the average expression of gene `i` for all genes. The red curve represents a smoothed lowess curve that has been fitted to reflect the overall trend of the data When the curve drops below `y = 0` (the blue line). we know that method `j` effects a greater reduction in the variation of the data relative to method `k`. Similarly, when the red curve is above `y = 0`, method `k` is more effective in reducing the variation. If the data from both methods have similar variances then the red curve should remain at `y = 0`.

The placement of the red curve below the horizontal axis indicates that quantile normalization produces a smaller variance than the housekeeping gene normalization on this example data set. This is the case across the entire spectrum of Ct values observed.

```
> plotVarMean(mynormQuant.data, mynormHK.data, normTag1 = "Quantile",
+     normTag2 = "HK-Gene")
```

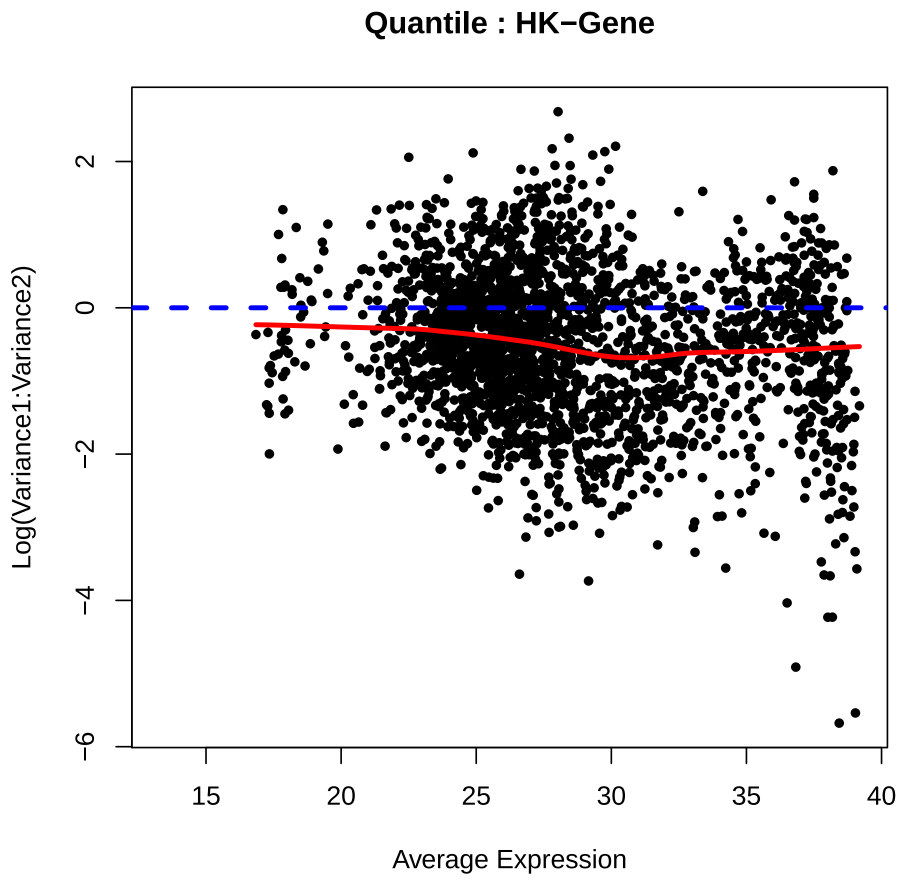

Figure 4: Variance Ratio versus Mean Plot Comparing Quantile Normalization with Housekeeping-Gene Normalization

5 More Info

For more information please see the documentation for the individual functions. Access to the technical report associated with the `qpcrNorm` package is available upon request (email `jess@jimmy.harvard.edu`).

6 Acknowledgements

We thank Dr Martin Aryee, Dr Jianhua Zhang and two anonymous reviewers for the valuable help and feedback that was instrumental to the creation of this package.

7 References

Bolstad BM, Irizarry RA, Astrand M, and Speed TP: A comparison of normalization methods for high density oligonucleotide array data based on bias and variance. Bioinformatics 2003, 19:185-193.

Mar JC, Kimura Y, Schroder K, Irvine KM, Hayashizaki Y, Suzuki H, Hume D, Quackenbush J: Data-driven normalization strategies for high-throughput quantitative RT-PCR. BMC Bioinformatics 2009, to appear.

Tseng GC, Oh MK, Rohlin L, Liao JC, Wong WH: Issues in cDNA microarray analysis: quality filtering, channel normalization, models of variations and assessment of gene effects. Nucleic Acids Research 2001, 29:2549-2557.
