# Supplementary material for: Data-driven normalization strategies for high-throughput quantitative RT-PCR
Source: BMC Bioinformatics. 2009 Apr 19;10:110. doi: 10.1186/1471-2105-10-110 (PMC2680405; doi:10.1186/1471-2105-10-110)
Supplement: Additional file 3 — Tutorial and qpcrNorm Software. The R package qpcrNorm and a tutorial outlining its use. [file 1471-2105-10-110-S3.zip › qpcrNorm_supplemental/qpcrNorm_1.0.0.zip!/qpcrNorm/doc/index.html]

R: qpcrNorm vignettes

## Vignettes of package qpcrNorm

qpcrNorm.pdf:: qPCR Normalization Example
