# Supplementary material for: Data-driven normalization strategies for high-throughput quantitative RT-PCR
Source: BMC Bioinformatics. 2009 Apr 19;10:110. doi: 10.1186/1471-2105-10-110 (PMC2680405; doi:10.1186/1471-2105-10-110)
Supplement: Additional file 3 — Tutorial and qpcrNorm Software. The R package qpcrNorm and a tutorial outlining its use. [file 1471-2105-10-110-S3.zip › qpcrNorm_supplemental/qpcrNorm_1.0.0.zip!/qpcrNorm/doc/Rplots.pdf]

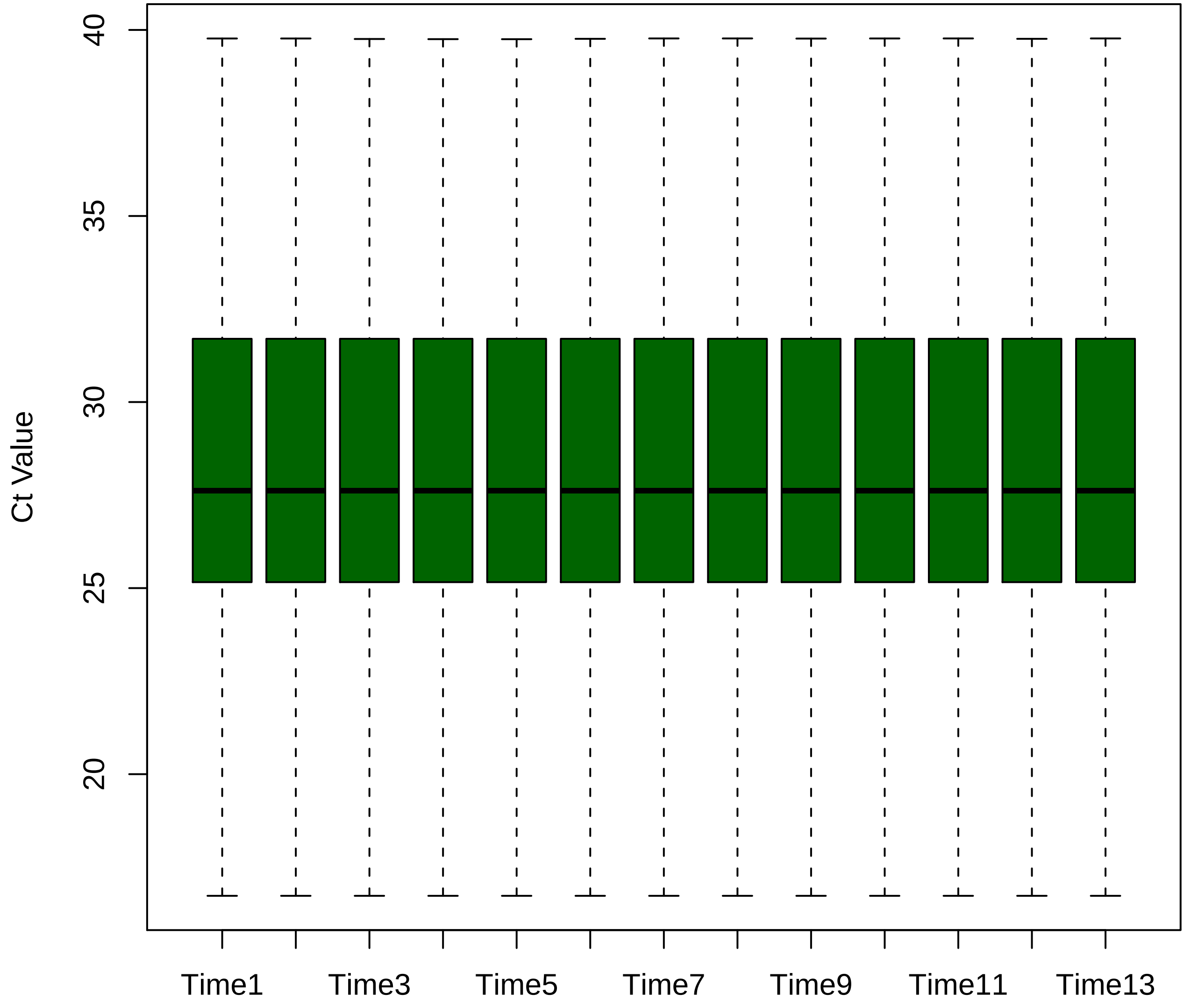
Ct Value
40
35
30
25
20
Time1
Time3
Time5
Time7
Time9
Time11
Time13

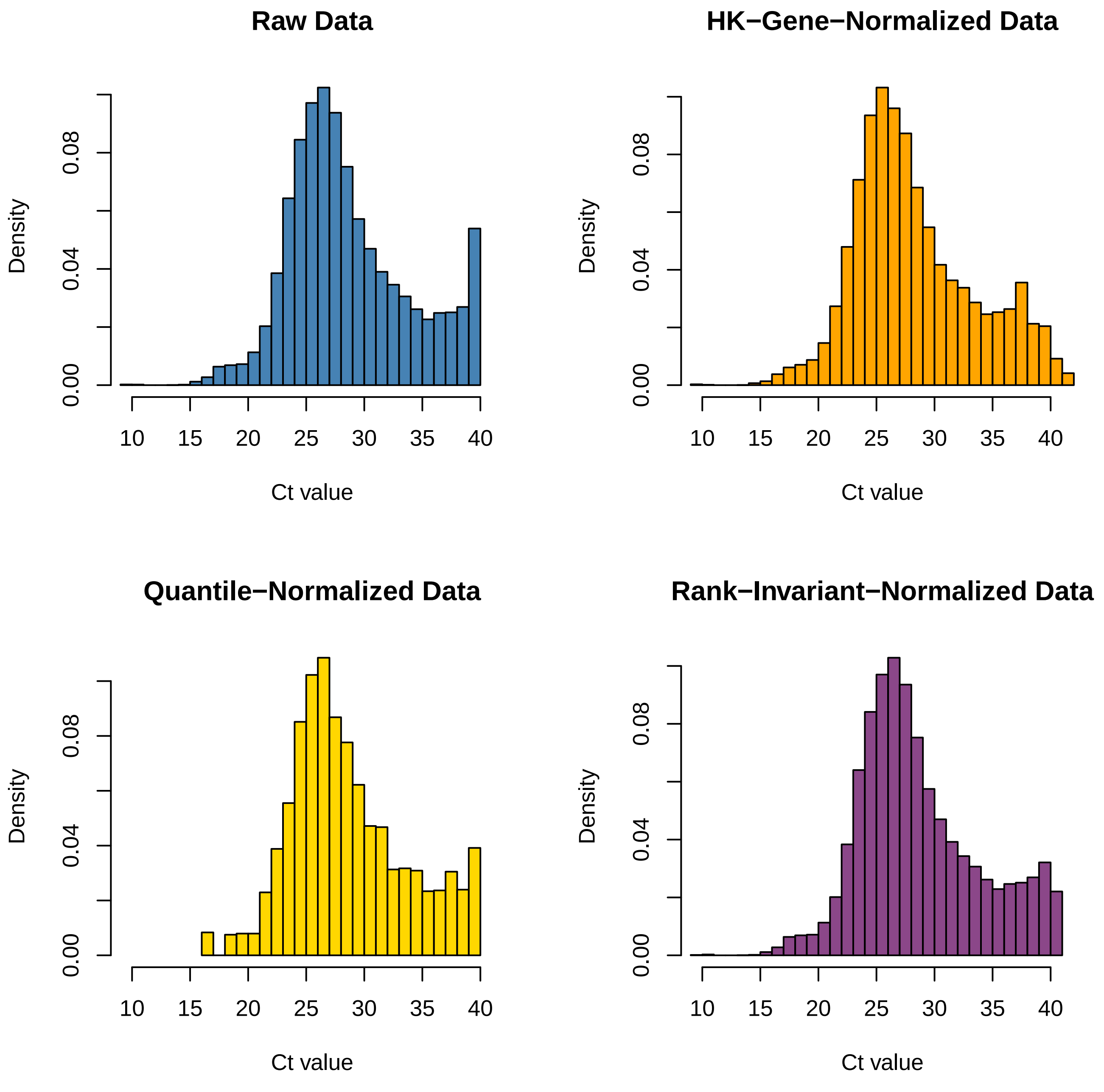

Raw Data
HK−Gene−Normalized Data
Quantile−Normalized Data
Rank−Invariant−Normalized Data
Density
0.00
0.04
0.08
Ct value
10
15
20
25
30
35
40

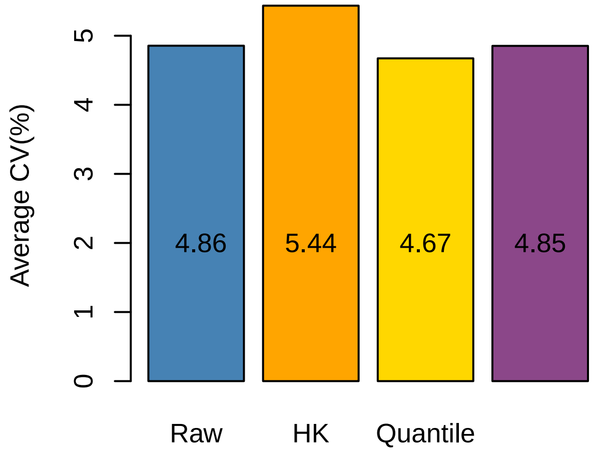
Average CV(%)
0
1
2
3
4
5
4.86
5.44
4.67
4.85
Raw
HK
Quantile

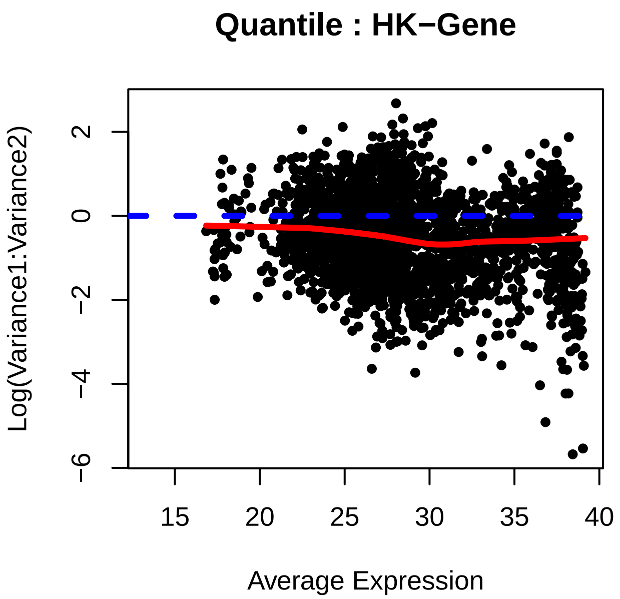
Quantile : HK−Gene
Log(Variance1:Variance2)
−6
−4
−2
0
2
15
20
25
30
35
40
Average Expression
