# Supplementary material for: Data-driven normalization strategies for high-throughput quantitative RT-PCR
Source: BMC Bioinformatics. 2009 Apr 19;10:110. doi: 10.1186/1471-2105-10-110 (PMC2680405; doi:10.1186/1471-2105-10-110)
Supplement: Additional file 3 — Tutorial and qpcrNorm Software. The R package qpcrNorm and a tutorial outlining its use. [file 1471-2105-10-110-S3.zip › qpcrNorm_supplemental/qpcrNorm_1.0.0.zip!/qpcrNorm/html/00Index.html]

R: Data-driven normalization strategies for high-throughput qPCR
data.

# Data-driven normalization strategies for high-throughput qPCR data.

---

## Documentation for package ‘qpcrNorm’ version 1.0.0

## User Guides and Package Vignettes

Read overview or browse directory.

## Help Pages

|  |  |
| --- | --- |
| calcCV | Calculates the Average Gene-Specific Coefficient of Variation |
| normalize, qpcrBatch-class | Class qpcrBatch |
| normQpcrHouseKeepingGenes | Function for Housekeeping Gene Normalization of qPCR Data. |
| normQpcrQuantile | Function for Quantile Normalization of qPCR Data. |
| normQpcrRankInvariant | Function for Rank-Invariant Set Normalization for qPCR Data. |
| plotVarMean | Constructs scatter plot to compare the effects of two normalization algorithms on a qPCR dataset. |
| qpcrBatch | Class qpcrBatch |
| qpcrBatch-class | Class qpcrBatch |
| qpcrBatch.object | qpcrBatch instance qpcrBatch.object |
| readQpcr | Data Input Function for a Single qPCR Experiment. |
| readQpcrBatch | Data Input Function for a Batch of qPCR Experiments. |
| writeQpcr | Writes qpcrBatch object out to a File. |
