# Supplementary material for: Data-driven normalization strategies for high-throughput quantitative RT-PCR
Source: BMC Bioinformatics. 2009 Apr 19;10:110. doi: 10.1186/1471-2105-10-110 (PMC2680405; doi:10.1186/1471-2105-10-110)
Supplement: Additional file 3 — Tutorial and qpcrNorm Software. The R package qpcrNorm and a tutorial outlining its use. [file 1471-2105-10-110-S3.zip › qpcrNorm_supplemental/qpcrNorm_1.0.0.zip!/qpcrNorm/html/calcCV.html]

R: Calculates the Average Gene-Specific Coefficient of Variation

|  |  |
| --- | --- |
| calcCV {qpcrNorm} | R Documentation |

## Calculates the Average Gene-Specific Coefficient of Variation

### Description

This function calculates the coefficient of variation for each gene in the qPCR experiment,
and returns the average coefficient of variation across all genes.

### Usage

```
calcCV(qBatch)
```

### Arguments

|  |  |
| --- | --- |
| `qBatch` | A `qpcrBatch` object. |

### Value

A `numeric` value.

### Author(s)

Jess Mar jess@jimmy.harvard.edu

### Examples

```
        data(qpcrBatch.object) 
        mynormRI.data <- normQpcrRankInvariant(qpcrBatch.object, 1)
        mynormQuant.data <- normQpcrQuantile(qpcrBatch.object)    
        barplot(c(calcCV(mynormRI.data), calcCV(mynormQuant.data)), col=c("red", "blue"))
```

---

[Package *qpcrNorm* version 1.0.0 Index]
