# Supplementary material for: Data-driven normalization strategies for high-throughput quantitative RT-PCR
Source: BMC Bioinformatics. 2009 Apr 19;10:110. doi: 10.1186/1471-2105-10-110 (PMC2680405; doi:10.1186/1471-2105-10-110)
Supplement: Additional file 3 — Tutorial and qpcrNorm Software. The R package qpcrNorm and a tutorial outlining its use. [file 1471-2105-10-110-S3.zip › qpcrNorm_supplemental/qpcrNorm_1.0.0.zip!/qpcrNorm/html/ctQc.html]

R: Quality Control Filter for Replicate Ct Values

|  |  |
| --- | --- |
| ctQc {qpcrNorm} | R Documentation |

## Quality Control Filter for Replicate Ct Values

### Description

This function applies a quality control filter to triplicate Ct values before
combining them into a single summary Ct measure.

Current implementation can only handle three replicates.

### Usage

```
ctQc(x)
```

### Arguments

|  |  |
| --- | --- |
| `x` | Matrix with three columns, corresponding to the triplicate Ct values. |

### Details

Applying ctQc is an alternative to averaging the triplicate Ct values. This filter was originally developed by Yasumasa Kimura
1. For each primer, sort Ct values in ascending order so we have [ct1, ct2, ct3].
2. Take two differences
difference1 := ct2 - ct1
difference2 := ct3 - ct2
If either or both have ct 40, we don't calculate the difference and set it to region 4 in the next step.
3. Take an average according to the differences.
Here we have 2 thresholds : 0.2 and 1.0. With the thresholds, we classify the 2 differences into the below regions.
region1 : difference <= 0.2
region2 : 0.2 < difference <= 1.0
region3 : 1.0 < difference
region4 : either or both ct are 40
If the 2 differences are in same region, we take an average of 3 ct values.
If the 2 differences are in different regions, we take an average of 2 ct values which are in smaller number region.

### Value

Numeric vector of Ct values combined over the three replicates according to the QC filter.

### Author(s)

Yasumasa Kimura   
Jess Mar jess@jimmy.harvard.edu

### See Also

`readQpcr`, `readQpcrBatch`

### Examples

```
        ## myQpcrBatch <- readQpcrBatch(qc=T)   # reads in data from a batch of qPCR experiments, applies ctQc to raw Ct values
```

---

[Package *qpcrNorm* version 1.0.0 Index]
