# Supplementary material for: Data-driven normalization strategies for high-throughput quantitative RT-PCR
Source: BMC Bioinformatics. 2009 Apr 19;10:110. doi: 10.1186/1471-2105-10-110 (PMC2680405; doi:10.1186/1471-2105-10-110)
Supplement: Additional file 3 — Tutorial and qpcrNorm Software. The R package qpcrNorm and a tutorial outlining its use. [file 1471-2105-10-110-S3.zip › qpcrNorm_supplemental/qpcrNorm_1.0.0.zip!/qpcrNorm/html/matrixByPlate.html]

R: Internal function to reorganize qPCR data into a rectangular structure.

|  |  |
| --- | --- |
| matrixByPlate {qpcrNorm} | R Documentation |

## Internal function to reorganize qPCR data into a rectangular structure.

### Description

This function takes a vector of Ct values from a single qPCR experiment and
reorganizes it into a matrix structure. Each column in the matrix represents a
different plate that was used in the experiment.

### Usage

```
matrixByPlate(xvec, plateIndex)
```

### Arguments

|  |  |
| --- | --- |
| `xvec` | Numeric vector of Ct values. |
| `plateIndex` | Character vector, denoting plate index of each gene or primer pair. |

### Details

On the resulting matrix structure:   
The number of rows equals the maximum number of genes or primer pairs that were used on a plate in the experiment.
For plates with less genes, NA values are padded at the end of the column vector to complete the rectangular structure.
Note: these NA values do not affect downstream calculations.

### Value

A `matrix` object.

### Author(s)

Jess Mar jess@jimmy.harvard.edu

### See Also

`normQpcrQuantile`

### Examples

```
        data(qpcrBatch.object)
        mynormQuant.data <- normQpcrQuantile(qpcrBatch.object)
```

---

[Package *qpcrNorm* version 1.0.0 Index]
