# Supplementary material for: Data-driven normalization strategies for high-throughput quantitative RT-PCR
Source: BMC Bioinformatics. 2009 Apr 19;10:110. doi: 10.1186/1471-2105-10-110 (PMC2680405; doi:10.1186/1471-2105-10-110)
Supplement: Additional file 3 — Tutorial and qpcrNorm Software. The R package qpcrNorm and a tutorial outlining its use. [file 1471-2105-10-110-S3.zip › qpcrNorm_supplemental/qpcrNorm_1.0.0.zip!/qpcrNorm/html/normQpcrHouseKeepingGenes.html]

R: Function for Housekeeping Gene Normalization of qPCR Data.

|  |  |
| --- | --- |
| normQpcrHouseKeepingGenes {qpcrNorm} | R Documentation |

## Function for Housekeeping Gene Normalization of qPCR Data.

### Description

Implements housekeeping gene normalization for a `qpcrBatch` object.

### Usage

```
normQpcrHouseKeepingGenes(qBatch, hkeep.genes)
```

### Arguments

|  |  |
| --- | --- |
| `qBatch` | A `qpcrBatch` object to be normalized. |
| `hkeep.genes` | Character vector, specifying which housekeeping genes to be used for normalization. |

### Details

The names in `hkeep.genes` must be a subset of the gene or primer pair names slot in the `qpcrBatch` object.

### Value

A `qpcrBatch` object, the `normalized` slot is now set at TRUE.

### Author(s)

Jess Mar jess@jimmy.harvard.edu

### See Also

`normQpcrQuantile`, `normQpcrRankInvariant`

### Examples

```
        data(qpcrBatch.object)
        mynormHK.data <- normQpcrHouseKeepingGenes(qpcrBatch.object, c("Gpx4"))
```

---

[Package *qpcrNorm* version 1.0.0 Index]
