# Supplementary material for: Data-driven normalization strategies for high-throughput quantitative RT-PCR
Source: BMC Bioinformatics. 2009 Apr 19;10:110. doi: 10.1186/1471-2105-10-110 (PMC2680405; doi:10.1186/1471-2105-10-110)
Supplement: Additional file 3 — Tutorial and qpcrNorm Software. The R package qpcrNorm and a tutorial outlining its use. [file 1471-2105-10-110-S3.zip › qpcrNorm_supplemental/qpcrNorm_1.0.0.zip!/qpcrNorm/html/normQpcrQuantile.html]

R: Function for Quantile Normalization of qPCR Data.

|  |  |
| --- | --- |
| normQpcrQuantile {qpcrNorm} | R Documentation |

## Function for Quantile Normalization of qPCR Data.

### Description

Implements quantile normalization for a `qpcrBatch` object.
We have adapted this algorithm from the function `normalizeBetweenArrays` from the limma package.
Data in a `qpcrBatch` object is normalized such that within an experiment, the expression distributions
across plates are more or less identical, and across experiments, the expression distributions
are also now more or less identical.

### Usage

```
normQpcrQuantile(qBatch)
```

### Arguments

|  |  |
| --- | --- |
| `qBatch` | A `link{qpcrBatch}` object. |

### Value

A `link{qpcrBatch}` object, the `normalized` slot is now set at TRUE.

### Author(s)

Jess Mar jess@jimmy.harvard.edu

### See Also

`normQpcrRankInvariant`, `normalizeBetweenArrays`

### Examples

```
 data(qpcrBatch.object) 
 mynormQuant.data <- normQpcrQuantile(qpcrBatch.object)
```

---

[Package *qpcrNorm* version 1.0.0 Index]
