# Supplementary material for: Data-driven normalization strategies for high-throughput quantitative RT-PCR
Source: BMC Bioinformatics. 2009 Apr 19;10:110. doi: 10.1186/1471-2105-10-110 (PMC2680405; doi:10.1186/1471-2105-10-110)
Supplement: Additional file 3 — Tutorial and qpcrNorm Software. The R package qpcrNorm and a tutorial outlining its use. [file 1471-2105-10-110-S3.zip › qpcrNorm_supplemental/qpcrNorm_1.0.0.zip!/qpcrNorm/html/normQpcrRankInvariant.html]

R: Function for Rank-Invariant Set Normalization for qPCR Data.

|  |  |
| --- | --- |
| normQpcrRankInvariant {qpcrNorm} | R Documentation |

## Function for Rank-Invariant Set Normalization for qPCR Data.

### Description

Implements rank-invariant set normalization for a `qpcrBatch` object.
We have adapted this algorithm from the function `normalize.invariantset`
from the affy package.

### Usage

```
normQpcrRankInvariant(qBatch, refType, rem.highCt = FALSE, thresh.Ct = 30)
```

### Arguments

|  |  |
| --- | --- |
| `qBatch` | A `qpcrBatch` object. |
| `refType` | Indicates what reference sample should be used, can be an integer or character string. See Details below. |
| `rem.highCt` | Logical indicator, TRUE if user wishes to remove genes with high Ct values (very low expression) that may be associated poor data quality. |
| `thresh.Ct` | Numerical value indicating the Ct value cutoff threshold, if `rem.highCt` = FALSE, genes with Ct values > thresh.Ct are removed from the data set. |

### Details

The algorithm computes all rank-invariant sets of genes between pairwise comparisons where each
experimental sample in the `qpcrBatch` object is paired against a reference. There are several ways to specify
what a sensible choice for the reference sample should be.   
  
1. The reference is an experimental sample in the `qpcrBatch` object.   
Specify `refType` as an `integer` value, corresponding to the index of which experimental sample is the reference.   
  
2. The reference is the sample which is closest to mean of all the experiments.   
Specify `refType = "mean"`.   
  
3. The reference is the sample which is closest to median of all the experiments.   
Specify `refType = "median"`.   
  
4. The reference is the mean of all experiments in the `qpcrBatch` object.   
Specify `refType = "pseudo.mean"`.   
  
5. The reference is the median of all experiments in the `qpcrBatch` object.   
Specify `refType = "pseudo.median"`.

### Value

A `qpcrBatch` object, the `normalized` slot is now set at TRUE.
The names of the rank-invariant genes used for normalization are stored as a vector in the `normGenes` slot of the `qpcrBatch` object returned.
To retrieve the rank-invariant gene names, use `qpcrBatch@normGenes`.

### Author(s)

Jess Mar jess@jimmy.harvard.edu

### See Also

`normQpcrQuantile`, `normalize.invariantset`

### Examples

```
 data(qpcrBatch.object)
 mynormRI.data <- normQpcrRankInvariant(qpcrBatch.object, 1) 
 mynormRI.data@normGenes                # retrieves names of genes in the rank-invariant set
```

---

[Package *qpcrNorm* version 1.0.0 Index]
