# Supplementary material for: Data-driven normalization strategies for high-throughput quantitative RT-PCR
Source: BMC Bioinformatics. 2009 Apr 19;10:110. doi: 10.1186/1471-2105-10-110 (PMC2680405; doi:10.1186/1471-2105-10-110)
Supplement: Additional file 3 — Tutorial and qpcrNorm Software. The R package qpcrNorm and a tutorial outlining its use. [file 1471-2105-10-110-S3.zip › qpcrNorm_supplemental/qpcrNorm_1.0.0.zip!/qpcrNorm/html/plotVarMean.html]

R: Constructs scatter plot to compare the effects of two normalization algorithms on a qPCR dataset.

|  |  |
| --- | --- |
| plotVarMean {qpcrNorm} | R Documentation |

## Constructs scatter plot to compare the effects of two normalization algorithms on a qPCR dataset.

### Description

This function makes a scatter plot which serves as a useful exploratory tool in evaluating whether one
normalization algorithm has been more effective than another on a given qPCR dataset.

### Usage

```
plotVarMean(qpcrBatch1, qpcrBatch2, normTag1 = "Normalization Type1", normTag2 = "Normalization Type2", ...)
```

### Arguments

|  |  |
| --- | --- |
| `qpcrBatch1` | A `qpcrBatch` object. |
| `qpcrBatch2` | A `qpcrBatch` object. |
| `normTag1` | Character string denoting what normalization algorithm was used for this data set. |
| `normTag2` | Character string denoting what normalization algorithm was used for this data set. |
| `...` | Further arguments can be supplied to the `plot` function. |

### Details

For each gene, the function plots its log-transformed ratio of its expression variance in
one normalized dataset versus another normalized dataset, i.e. let Gij be the variance of
the expression values of gene i that have been normalized with method j.
We plot the natural log-transformed ratio of Gij to Gik on the y-axis,
and the average expression of gene i on the x-axis for all genes. /cr
The red curve represents a smoothed lowess curve that has been fitted to reflect the overall
trend of the data. When the red curve drops below y = 0 (the blue dotted line) we know that method j
effects a greater reduction in the variation of the data over method k.
Similarly, when the red curve is above y = 0, method k is more effective in reducing the variation
in the data than method j. If the data from both methods have similar variances then the red curve
should remain at y = 0. Bolstad et al. (2003) originally used these plots for variance comparisons of
different normalization methods for high density oligonucleotide array data.

### Value

A `plot` object.

### Author(s)

Jess Mar jess@jimmy.harvard.edu

### References

Bolstad B et al. A comparison of normalization methods for high density oligonucleotide array data based on variance and bias. Bioinformatics, 2003.

### See Also

`plot`

### Examples

```
  # data(qpcrBatch.object)
  # mynormRI.data <- normQpcrRankInvariant(qpcrBatch.object, 1) 
  # mynormQuant.data <- normQpcrQuantile(qpcrBatch.object)
  # plotVarMean(mynormRI.data, mynormQuant.data, normTag1="Rank-Invariant", normTag2="Quantile", main="Comparing Two Data-driven Methods")
```

---

[Package *qpcrNorm* version 1.0.0 Index]
