# Supplementary material for: Data-driven normalization strategies for high-throughput quantitative RT-PCR
Source: BMC Bioinformatics. 2009 Apr 19;10:110. doi: 10.1186/1471-2105-10-110 (PMC2680405; doi:10.1186/1471-2105-10-110)
Supplement: Additional file 3 — Tutorial and qpcrNorm Software. The R package qpcrNorm and a tutorial outlining its use. [file 1471-2105-10-110-S3.zip › qpcrNorm_supplemental/qpcrNorm_1.0.0.zip!/qpcrNorm/html/qpcrBatch-class.html]

R: Class qpcrBatch

|  |  |
| --- | --- |
| qpcrBatch-class {qpcrNorm} | R Documentation |

## Class qpcrBatch

### Description

This is a class representation for qPCR expression data.

### Objects from the Class

Objects can be created using the function `readQpcr` or `readQpcrBatch` to read in
raw data from a text file(s). Objects can also be created by using `new("qpcrBatch", ...)`.

### Slots

`geneNames`:
:   Character vector denoting gene or primer pair names.

`plateIndex`:
:   Character vector denoting plate indices.

`exprs`:
:   Matrix of qPCR expression values, normally these are the Ct values.

`normalized`:
:   Logical value, TRUE if expression data has been normalized.

`normGenes`:
:   Character vector of genes used by the normalization algorithm.

### Methods

No methods have yet been defined with class "qpcrBatch" in the signature.

### Note

This class is better describe in the vignette.

### Author(s)

Jess Mar jess@jimmy.harvard.edu

### Examples

```
## load example data 
data(qpcrBatch.object)
class(qpcrBatch.object)
```

---

[Package *qpcrNorm* version 1.0.0 Index]
