# Supplementary material for: Data-driven normalization strategies for high-throughput quantitative RT-PCR
Source: BMC Bioinformatics. 2009 Apr 19;10:110. doi: 10.1186/1471-2105-10-110 (PMC2680405; doi:10.1186/1471-2105-10-110)
Supplement: Additional file 3 — Tutorial and qpcrNorm Software. The R package qpcrNorm and a tutorial outlining its use. [file 1471-2105-10-110-S3.zip › qpcrNorm_supplemental/qpcrNorm_1.0.0.zip!/qpcrNorm/html/qpcrBatch.object.html]

R: qpcrBatch instance qpcrBatch.object

|  |  |
| --- | --- |
| qpcrBatch.object {qpcrNorm} | R Documentation |

## qpcrBatch instance qpcrBatch.object

### Description

This is an artifically generated qPCR data set. The data set
has been closely simulated from original data for 2396 genes on 13 time points.
Each measurement within the one sample was repeated over three replicate wells,
across multiple plates.

### Usage

```
data(qpcrBatch.object)
```

### Format

A data frame with 2396 observations on the following 41 variables.

`Primers`
:   Character vector of gene or primer pair names.

`Plate_Index`
:   Numeric vector denoting plate indices.

`Time1_Rep1`
:   Ct values for first time point, first replicate.

`Time1_Rep2`
:   Ct values for first time point, second replicate.

`Time1_Rep3`
:   Ct values for first time point, third replicate.

### Examples

```
data(qpcrBatch.object)
```

---

[Package *qpcrNorm* version 1.0.0 Index]
