# Supplementary material for: Data-driven normalization strategies for high-throughput quantitative RT-PCR
Source: BMC Bioinformatics. 2009 Apr 19;10:110. doi: 10.1186/1471-2105-10-110 (PMC2680405; doi:10.1186/1471-2105-10-110)
Supplement: Additional file 3 — Tutorial and qpcrNorm Software. The R package qpcrNorm and a tutorial outlining its use. [file 1471-2105-10-110-S3.zip › qpcrNorm_supplemental/qpcrNorm_1.0.0.zip!/qpcrNorm/html/readQpcr.html]

R: Data Input Function for a Single qPCR Experiment.

|  |  |
| --- | --- |
| readQpcr {qpcrNorm} | R Documentation |

## Data Input Function for a Single qPCR Experiment.

### Description

This function reads in data from a single qPCR experiment. The text file must have the following structure:   
1st column = names denoting genes or primer pairs   
2nd column = plate index of each gene or primer pair   
remaining columns = (replicate) Ct values.   
Note: other annotation fields may be added after the Ct value columns.

### Usage

```
readQpcr(fileName, header = TRUE, qc = FALSE, quote = "\"", dec = ".", fill = TRUE, comment.char = "", ...)
```

### Arguments

|  |  |
| --- | --- |
| `fileName` | Character string. |
| `header` | Logical value, TRUE if the file contains the names of the variables as its first line. |
| `qc` | Logical value, TRUE if a QC filter `ctQc` should be applied to the data.   If qc = F, the replicate Ct values will be averaged. |
| `quote` | Set of quoting characters. To disable quoting, set quote = "". See `scan` for behaviour on quotes embedded in quotes. |
| `dec` | Character used for decimal points. |
| `fill` | Logical value, TRUE if in case rows have unequal length, blank fields are implicitly added. See `read.table`. |
| `comment.char` | Character vector of length one containing a single character or an empty string. Use "" to turn off the interpretation of comments altogether. |
| `...` | further arguments to be passed to `read.table`. |

### Details

Currently fields appearing after the Ct value columns are not associated with the output of this function.
Note: the majority of arguments to readQpcr are identical to those supplied to read.table. These have been included to
give the user greater control over data input, should the data deviate from a standard tab-delimited file structure.
For a standard tab-delimited text file, specifying the `fileName` should be sufficient.

### Value

A `qpcrBatch` object.

### Author(s)

Jess Mar jess@jimmy.harvard.edu

### See Also

`readQpcrBatch`, `ctQc`

### Examples

```
        ## onerun.data <- readQpcr("singleQpcrRun.txt")
```

---

[Package *qpcrNorm* version 1.0.0 Index]
