# Supplementary material for: Data-driven normalization strategies for high-throughput quantitative RT-PCR
Source: BMC Bioinformatics. 2009 Apr 19;10:110. doi: 10.1186/1471-2105-10-110 (PMC2680405; doi:10.1186/1471-2105-10-110)
Supplement: Additional file 3 — Tutorial and qpcrNorm Software. The R package qpcrNorm and a tutorial outlining its use. [file 1471-2105-10-110-S3.zip › qpcrNorm_supplemental/qpcrNorm_1.0.0.zip!/qpcrNorm/html/readQpcrBatch.html]

R: Data Input Function for a Batch of qPCR Experiments.

|  |  |
| --- | --- |
| readQpcrBatch {qpcrNorm} | R Documentation |

## Data Input Function for a Batch of qPCR Experiments.

### Description

This function reads in data from multiple qPCR experiments from the one batch.
Each text file in the batch must meet the structure required by `readQpcr`.   
Note: In order to qualify as a batch, it is assumed that the same set of primers
are being analyzed in each experiment.

### Usage

```
readQpcrBatch(..., filenames = character(), qc = FALSE)
```

### Arguments

|  |  |
| --- | --- |
| `...` | Filenames separated by a comma. |
| `filenames` | Character vector specifying file names. |
| `qc` | Logical value, TRUE if a QC filter `ctQc` should be applied to the data.   If qc = F, the replicate Ct values will be averaged. See `ctQc`. |

### Details

If the function is called with no arguments `readQpcrBatch()` all the files in the working directory are
read and put into a `qpcrBatch` object.
All files must conform to the following structure:   
1st column = names denoting genes or primer pairs   
2nd column = plate index of each gene or primer pair   
remaining columns = (replicate) Ct values   
other annotation fields may be added after the Ct value columns. /cr /cr

Currently fields appearing after the Ct value columns are not associated with the output of this function.
Note: the majority of arguments to readQpcr are identical to those supplied to read.table. These have been included to
give the user greater control over data input, should the data deviate from a standard tab-delimited file structure.
For a set of standard tab-delimited text files, specifying the `filenames` should be sufficient.

### Value

A `qpcrBatch` object.

### Author(s)

Jess Mar jess@jimmy.harvard.edu

### See Also

`ctQc`, `readQpcr`, `setwd`

### Examples

```
        ## myBatch <- readQpcrBatch()
```

---

[Package *qpcrNorm* version 1.0.0 Index]
