# Supplementary material for: Data-driven normalization strategies for high-throughput quantitative RT-PCR
Source: BMC Bioinformatics. 2009 Apr 19;10:110. doi: 10.1186/1471-2105-10-110 (PMC2680405; doi:10.1186/1471-2105-10-110)
Supplement: Additional file 3 — Tutorial and qpcrNorm Software. The R package qpcrNorm and a tutorial outlining its use. [file 1471-2105-10-110-S3.zip › qpcrNorm_supplemental/qpcrNorm_1.0.0.zip!/qpcrNorm/html/writeQpcr.html]

R: Writes qpcrBatch object out to a File.

|  |  |
| --- | --- |
| writeQpcr {qpcrNorm} | R Documentation |

## Writes qpcrBatch object out to a File.

### Description

This function writes a `qpcrBatch` out to a tab-delimited text file.
`writeQpcr` can be used to write out the normalized qPCR data out
to an external file.

### Usage

```
  writeQpcr(qBatch, fileName, ...)
```

### Arguments

|  |  |
| --- | --- |
| `qBatch` | A `qpcrBatch` object. |
| `fileName` | Character string specifying name of the output file. |
| `...` | Extra arguments to be passed to `write.table`. |

### Details

Function creates a tab-delimited text file with three columns,   
1st column = names denoting genes or primer pairs
2nd column = plate index
3rd column = normalized Ct value

### Author(s)

Jess Mar jess@jimmy.harvard.edu

### References

Mar J et al. Data-driven Normalization Strategies for qPCR Data. Technical Report, 2008.

### See Also

`write.table`

### Examples

```
        ## writeQpcr(qpcrBatch.object, "output1.txt")
```

---

[Package *qpcrNorm* version 1.0.0 Index]
